# External validation of the Simplified PADUA REnal (SPARE) nephrometry system in predicting surgical outcomes after partial nephrectomy

**DOI:** 10.1186/s12894-020-00702-6

**Published:** 2020-09-11

**Authors:** Chi-Ping Huang, Chao-Hsiang Chang, Hsi-Chin Wu, Che-Rei Yang, Po-Fan Hsieh, Guang-Heng Chen, Po-Jen Hsiao, Yi-Huei Chang, Yu-Ping Wang, Yu-De Wang

**Affiliations:** 1 Department of Urology, China Medical University Hospital, No. 2, Yu-De Rd., Taichung City, Taiwan (R.O.C.); 2 School of Medicine, China Medical University, No. 91, Xueshi Rd., North Dist., Taichung City, 404 Taiwan (R.O.C.); 3 Department of Urology, China Medical University Beigang Hospital, No. 123, Xinde Rd., Beigang Township, Yunlin County, 651 Taiwan (R.O.C.); 4 Department of Urology, China Medical University Hsinchu Hospital, No. 199, Sec. 1, Xinglong Rd., Zhubei City, Hsinchu County 302 Taiwan (R.O.C.); 5 Department of Radiology, Taichung Veterans General Hospital, No. 1650, Sec. 4, Taiwan Blvd., Xitun Dist., Taichung City, 407 Taiwan (R.O.C.)

**Keywords:** SPARE system, Partial nephrectomy, Pentafecta, Nephrometry

## Abstract

**Background:**

Pentafecta is a major goal in the era of partial nephrectomy (PN). Simplified PADUA REnal (SPARE) nephrometry system was developed to evaluate the complexity of tumor. However, the predictive ability in pentafecta of SPARE system is yet to be determined. The aim of this study was to externally validate the applicability of SPARE nephrometry system in predicting pentafecta achievement after partial nephrectomy, and to examine inter-observer concordance.

**Methods:**

We retrospectively reviewed data of 207 consecutive patients who underwent PN between January 2012 and August 2018 at a tertiary referral center. We obtained SPARE, R.E.N.A.L., and PADUA scores and evaluated correlations among the nephrometries and surgical outcomes including pentafecta by Spearman test. Logistic regression analysis was used to identify independent predictors of pentafecta outcomes. We compared the nephrometries to determine the predictive ability of achieving pentafecta using receiver operating characteristic curve analysis. Fleiss’ generalized kappa was used to assessed interobserver variation in the SPARE system.

**Results:**

Based on the SPARE system, 120, 74, and 13 patients were stratified into low-risk, intermediate-risk, and high-risk groups, respectively. Regarding the individual components of pentafecta, there were significant differences in the complication rate (*p* = 0.03), ischemia time (*p* < 0.001), and percent change of eGFR (*p* < 0.001) among the three risk groups. In addition, higher tumor complexity was significantly associated with a lower achievement rate of pentafecta (*p* = 0.01). In Spearman correlation tests, SPARE nephrometry was correlated with ischemia time (ρ:0.37, *p* < 0.001), operative time (ρ:0.28, *p* < 0.001), complication rate (ρ:0.34, *p* < 0.001), percent change of eGFR (ρ:0.34, *p* < 0.001), and progression of chronic kidney disease stage (ρ:0.17, *p* = 0.02). Multivariate analysis revealed that SPARE significantly affected pentafecta (OR: 0.67, *p* < 0.001). In ROC curve analysis, SPARE showed fair predictive ability in the achievement pentafecta (AUC: 0.71). The predictive ability of pentafecta was similar between nephrometries (SPARE vs. R.E.N.A.L., *p* = 0.78; SPARE vs. PADUA, *p* = 0.66). The interobserver concordance of SPARE was excellent (Kappa: 0.82, *p* = 0.03).

**Conclusions:**

SPARE system was a predictive factor of surgical outcomes after PN. This refined nephrometry had similar predictive abilities for pentafecta achievement compared with R.E.N.A.L. and PADUA.

## Background

Partial nephrectomy (PN) is the standard of care despite the increased use of surgical approaches for T1 renal tumors and even selected T2 renal tumors [1]. Compared to radical nephrectomy, PN can achieve better renal function preservation without compromising the oncological and overall survival outcomes [2, 3]. Both trifecta and pentafecta remain the major goals in the era of PN [4, 5]. Trifecta is an evaluation of short-term outcomes and is defined as ischemia time ≤ 25 min, negative surgical margin, and no major complications (defined as a Clavien score of ≧3). Pentafecta is an evaluation of long-term outcomes, that includes all of the criteria of trifecta in addition to including > 90% preservation of estimated glomerular filtration rate (eGFR) and no increase in the stage of chronic kidney disease (CKD) at 1 year after PN. These surgical outcomes are impacted by factors including patient characteristics and tumor complexity [6]. Therefore, standard, reproducible, and precise evaluations of tumor complexity are important in surgical planning and patient counseling.

**Fig. 1:**
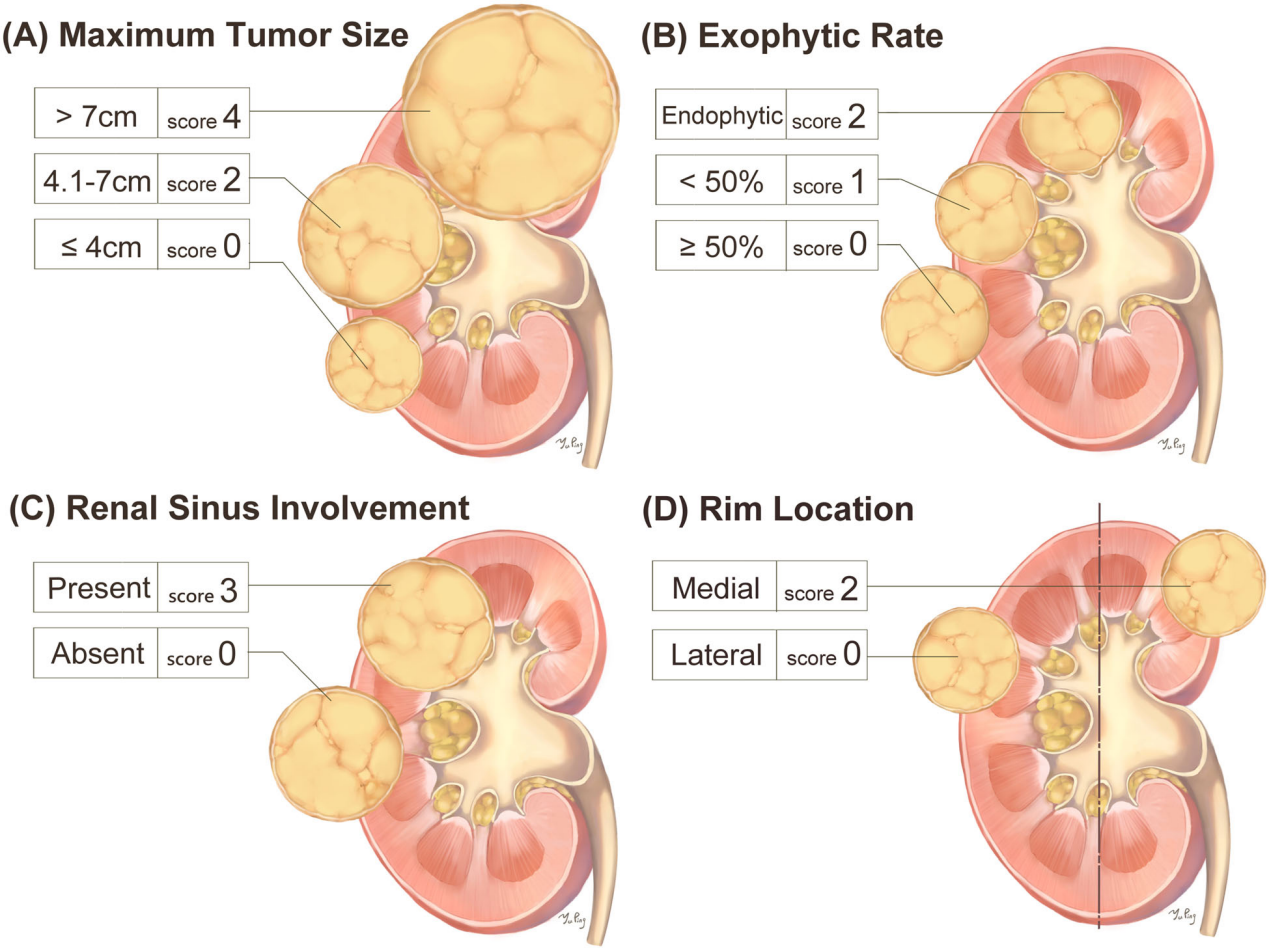
SPARE system (**a**) tumor size classification, (**b**) tumor deepening into the parenchyma, (**c**) tumor relationship with renal sinus, (**d**) margin location of the tumor; This figure is created by YPW

Several nephrometries have been developed and evaluated, of which the R.E.N.A.L. and PADUA systems are the most widely used and studied [7, 8]. Both R.E.N.A.L. and PADUA have been significantly correlated with prolonged ischemia time and post-operative complications, which are the component of trifecta [9]. However, controversy exists with regards to the application of these first generation nephrometries in the prediction of post-operative renal function, which are the component of pentafecta [10, 11]. Only the radius of the tumor and endophytic features are associated with split renal function after PN. Many factors in first generation nephrometries may decrease their predictive ability of functional outcomes [12]. The evolution of surgical techniques and the increasing use of PN may limit the use of first generation nephrometries. Ficarra et al. proposed a revised version of PADUA, the Simplified PADUA REnal (SPARE) nephrometry system [13]. The SPARE system is composed of fewer variables, including: 1) rim location; 2) renal sinus involvement; 3) exophytic rate, and 4) tumor size (Fig. 1). Even though fewer variables are used in the SPARE system, this has not negatively affected the ability to evaluate surgical complexity, and the accuracy to predict overall complications between the original PADUA and SPARE has been shown to be similar [13].

Since the SPARE system is a novel tool, its application and inter-observer concordance have yet to be validated externally. Moreover, few studies have evaluated the predictive ability of pentafecta between the SPARE system and first generation nephrometries. Therefore, the aim of this study was to apply three nephrometries (SPARE, R.E.N.A.L., PADUA) in a contemporary series of PNs in order to externally validate the SPARE system and a perform head-to-head comparisons of the predictive performance.

## Methods

### Patients and data collection

After Institutional Review Board (IRB) of China Medical University & Hospital approval (CMUH108-REC3–063), 207 consecutive patients who underwent PN via open, laparoscopic or robotic-assisted approaches for localized renal tumors between January 2012 and August 2018 at a tertiary referral center were included in this study. All methods were performed in accordance with the relevant guidelines and regulations, and a waiver of informed consent was granted by the IRB. Patients with multiple renal tumors within one kidney, solitary kidney, or recurrent renal cell carcinoma were excluded. The decision of surgical approach and technique of renorrhaphy were determined by the surgeons’ expertise and patients’ preference. All PNs were conducted by the standard renal artery and renal vein on-clamp technique, and conventional resection.

Image study with either abdominal computed tomography (CT) or magnetic resonance imaging (MRI) were obtained from all patients pre-operatively. Warm ischemia was used in LPN and RPN, and cold ischemia was used in OPN. We collected the patients’ demographic and clinical data and imaging studies electronically and analyzed them retrospectively. SPARE, R.E.N.A.L., and PADUA scores were obtained according to the original studies [7, 8, 13]. Based on risk stratification of the SPARE nephrometry, the patients were divided into three groups: low-risk group (score 0–3), intermediate-risk group (score 4–7), and high-risk group (score 8–10). Interobserver concordance of the SPARE nephrometry was assessed by two urologists and one radiologist (C.G. Heng, P.J. Hsiao, Y.P. Wang), each of whom was blinded to the clinical outcomes.

### Outcome measures

We collected and analyzed preoperative demographics (gender, age, American Society of Anesthesiologists score, Charlson Comorbidity Index), and perioperative outcomes (operative time, ischemia time, estimated blood loss, complications, length of hospitalization). Complications were defined as surgical-related adverse events within 3 months after surgery, and were assessed using the Clavien-Dindo classification system. A major complication was defined as a Clavien score of ≥3. Renal function was assessed by serum Cre and eGFR based on the Chronic Kidney Disease Epidemiology Collaboration (CKD-EPI) equation. Timing of renal function evaluation were pre-operatively, 3rd day, 30th day, and 1 year after surgery. Functional change in renal function was displayed in the absolute change of eGFR (ACE) and percent change of eGFR (PCE). CKD upstaging was defined as upstaging of CKD status to stage III, IV, or V. The following pathology features were recorded: malignancy, the subtype of RCC, and surgical margin. Pentafecta was assessed as previously reported [5].

### Statistical analyses

Categorical variables including sex, positive surgical margin, and achievement of pentafecta are displayed as a percentage. And continuous variables including SPARE, R.E.N.A.L. and PADUA scores are displayed as median (IQR). The Mann-Whitney U-test and Kruskal-Wallis H-test were used to compare two or more nonparametric continuous variables, respectively. The Pearson chi-square test was used to compare categorical variables. Spearman correlation was used to evaluate relationships among SPARE, R.E.N.A.L., and PADUA scores and surgical outcomes. Univariate and multivariate analyses between various clinical features including nephrometries and pentafecta were evaluated using a logistic regression model. Factors associated with pentafecta such as age, sex, ASA, CCI, BMI, hypertension, diabetes, pre-operative eGFR, surgical approach, SPARE, R.E.N.A.L., PADUA were included in univariate analysis. The variables with a *P*-value below 0.25 in the univariable models were used in subsequent multivariable models, each with a different scoring system locked in, which used a backward stepwise multivariable model selection process with a *P*-value threshold of 0.05 for variables to remain in the model. Since three nephrometry scores (SPARE, R.E.N.A.L., and PAUDA) are similar proxy variables for tumor complexity and correlating with each other. This method starts with all variables in the model and removes nonsignificant variables as well as those whose loss has negligible effect on the fit of the model. Three models were run for predicting each pentafecta individually. Each model made use of a different scoring system locked into the multivariate logistic regression model. The predictive abilities of the nephrometries for pentafecta were evaluated and compared using ROC curve analysis. We assessed interobserver variation in the SPARE system according to Fleiss’ generalized kappa. All analyses were performed using SPSS v.22 (SPSS, Chicago, IL, USA), and a *P* value < 0.05 was considered to be statistically significant.

## Results

Based on the SPARE system, 120, 74, and 13 patients were stratified into low-risk, intermediate-risk, and high-risk groups, respectively. There were no significant differences among the three groups in baseline characteristics except for tumor size (*p* < 0.001), surgical approach (*p* = 0.03), and tumor complexity assessed by the three nephrometries (p < 0.001) (Table 1). There was a trend that robotic surgery was preferred to the other two operative approaches in the high-risk group. Forty-eight, 52, and 107 patients underwent PN via open, laparoscopic, and robotic approaches respectively, of whom 50% were male. The median (IQR) age was 58 (15) years, the American Society of Anesthesiologists score was 2 (1), the Charlson Comorbidity Index score was 2 (3), and median (IQR) tumor size was 3.5 (1.9) cm.

**Table 1:** Demographic information

	Total	Low risk (0–3)	Intermediate risk (4–7)	High risk (8–10)	*P-value*
No.	207	120	74	13	
Age, years	58 (15)	58 (16)	56 (17.3)	54 (15)	0.64
Male gender	104 (50.2)	58 (48.3)	36 (48.6)	10 (76.9)	0.15
ASA	2 (1)	2 (1)	2 (1)	2 (0)	0.28
CCI	2 (3)	3 (2)	2 (3)	1 (4)	0.09
BMI, kg/m^2^	25.6 (5.8)	26.1 (5.9)	25.1 (5.9)	22.6 (6.9)	0.16
Hypertension	92 (44.4)	47 (39.2)	39 (52.7)	6 (46.2)	0.21
Diabetes	32 (15.5)	19 (15.8)	11 (14.9)	2 (15.4)	0.42
Tumor size, cm	3.5 (1.9)	3.2 (1.6)	3.9 (2.6)	6.1 (3.1)	< 0.001
Surgical approach					0.03
open	48 (23.2)	19 (15.8)	27 (36.5)	2 (15.4)	
laparoscopic	52 (25.1)	35 (29.2)	15 (20.3)	2 (15.4)	
robotic	107 (51.7)	66 (55)	32 (43.2)	9 (69.2)	
R.E.N.A.L.	7 (3)	6 (2)	7 (2.5)	8 (2)	< 0.001
PADUA	9 (2)	8 (2)	10 (2.5)	12 (1)	< 0.001
SPARE	3 (4)	2 (2)	5 (2.25)	8 (1)	< 0.001

Data are expressed as median (IQR), or n (%)

*ASA* American Society of Anesthesiologists score, *CCI* Charlson Comorbidity Index, *BMI* body mass index

The median (IQR) operative time was 227 (97) minutes, the ischemia time was 24 (11) minutes, the estimated blood loss was 150 (250) mL, and the length of hospital stay was 8 (2) days (Table 2). Peri-operative outcomes were significantly different in the three risk groups. The patients with a higher tumor risk had the longest operative time (*p* = 0.003) and the longest hospital stay (*p* = 0.02) (Table 2). Clear cell renal cell carcinoma (RCC) (45.9%) was the most common malignant tumor, followed by papillary RCC (8.7%) and chromophobic RCC (8.2%) (Table 2). Regarding the individual components of pentafecta, there were significant differences in the complication rate (*p* = 0.03), ischemia time (*p* < 0.001), and PCE (*p* < 0.001) among the three risk groups. In addition, higher tumor complexity was significantly associated with a lower achievement rate of pentafecta (*p* = 0.01) (Table 2).

**Table 2:** Peri-operative outcomes

	Total	Low risk (0–3)	Intermediate risk (4–7)	High risk (8–10)	*P-value*
Ischemia time, minutes	24 (11)	22 (10)	27 (13)	28 (13)	< 0.001
Operative time, minutes	227 (97)	210 (109)	234 (80.5)	265 (67)	0.003
EBL, mL	150 (250)	100 (250)	150 (212.5)	200 (175)	0.59
Complications					0.03
minor, Clavien-Dindo grade 2 or less	49 (23.7)	28 (23.3)	18 (24.3)	3 (23)	
major, Clavien-Dindo grade 3 or more	8 (3.9)	7 (5.8)	0 (0)	1 (7.7)	
Length of stay, days	8 (2)	7 (3)	8 (2)	8 (2.5)	0.02
ACE-3rd day	11 (21)	8.5 (21)	13.5 (24.25)	20 (30)	0.008
PCE-3rd day	14 (23)	10.5 (23)	17.5 (26.75)	22 (30)	0.005
ACE-30th day	8 (15)	4 (16)	9.5 (14)	17 (11)	0.007
PCE-30th day	10 (17)	6.5 (18)	11.5 (13.5)	16 (12.5)	0.018
ACE-1st year	12 (18)	8.5 (16)	14.5 (17.25)	29 (18.5)	< 0.001
PCE-1st year	14.9 (18.2)	11.8 (18.8)	17 (16.5)	34 (16.8)	< 0.001
CKD upstaging	30 (14.5)	16 (13.3)	10 (13.5)	4 (30.8)	0.22
Pathological features					0.45
clear cell RCC	95 (45.9)	52 (43.3)	37 (50)	6 (46.2)	
papillary RCC	18 (8.7)	10 (8.3)	7 (9.5)	1 (7.7)	
chromophobe RCC	17 (8.2)	7 (5.8)	9 (12.2)	1 (7.7)	
others	7 (3.4)	3 (2.5)	3 (4.1)	1 (7.7)	
oncocytoma	8 (3.9)	3 (2.5)	4 (5.4)	1 (7.7)	
angiomyolipoma	62 (30)	45 (37.5)	14 (18.9)	3 (23.1)	
Positive surgical margin	8 (3.9)	6 (5)	1 (1.4)	1 (7.7)	0.24
Achievement of trifecta	112 (54.1)	73 (60.8)	35 (47.3)	3 (23.1)	0.02
Achievement of pentafecta	51 (24.6)	43 (35.8)	8 (10.8)	0 (0)	0.01

Data are expressed as median (IQR), or n (%)

*EBL* estimated blood loss, *ACE* absolute change of estimated glomerular filtration rate, *PCE* percent change of estimated glomerular filtration rate, *CKD* chronic kidney disease, *RCC* renal cell carcinoma

Spearman correlation analysis showed that the three nephrometry systems were significant correlated with each other (*p* < 0.001) (Table 3). The SPARE system was correlated with ischemia time (ρ:0.37, *p* < 0.001), operative time (ρ:0.28, *p* < 0.001), complication rate (ρ:0.34, *p* < 0.001), length of stay (ρ:0.18, 0.009), PCE-1st year (ρ:0.34, *p* < 0.001), rate of increase in CKD stage (ρ:0.17, *p* = 0.01), and rate of achieving pentafecta (ρ: − 0.35, *p* < 0.001). The correlation between peri-operative outcomes and PADUA was similar to the SPARE system, while R.E.N.A.L. was also correlated with EBL (ρ:0.15, *p* = 0.03) additionally (Table 3).

**Table 3:** Correlation between nephrometries and peri-operative features

Variables	SPARE		R.E.N.A.L.		PADUA	
	Coefficient	*P-value*	Coefficient	*P-value*	Coefficient	*P-value*
SPARE			0.61	< 0.001	0.79	< 0.001
R.E.N.A.L.	0.61	< 0.001			0.61	< 0.001
PADUA	0.79	< 0.001	0.84	< 0.001		
Ischemia time (minutes)	0.37	< 0.001	0.38	< 0.001	0.4	< 0.001
Operative time (minutes)	0.28	< 0.001	0.23	< 0.001	0.25	< 0.001
EBL (mL)	0.11	0.11	0.15	0.03	0.13	0.07
Complications (Clavien-Dindo classification)	0.34	< 0.001	0.22	0.002	0.28	< 0.001
Length of stay (days)	0.18	0.009	0.13	0.06	0.16	0.03
PCE-1st year (%)	0.34	< 0.001	0.28	< 0.001	0.32	< 0.001
CKD upstaging (%)	0.17	0.01	0.23	0.01	0.24	< 0.001
Positive margin (%)	0.07	0.41	0.04	0.68	−0.03	0.7
Achievement of pentafecta (%)	−0.35	< 0.001	−0.29	0.001	−0.33	< 0.001

*EBL* estimated blood loss, *PCE* percent change of estimated glomerular filtration rate, *CKD* chronic kidney disease

**Fig. 2:**
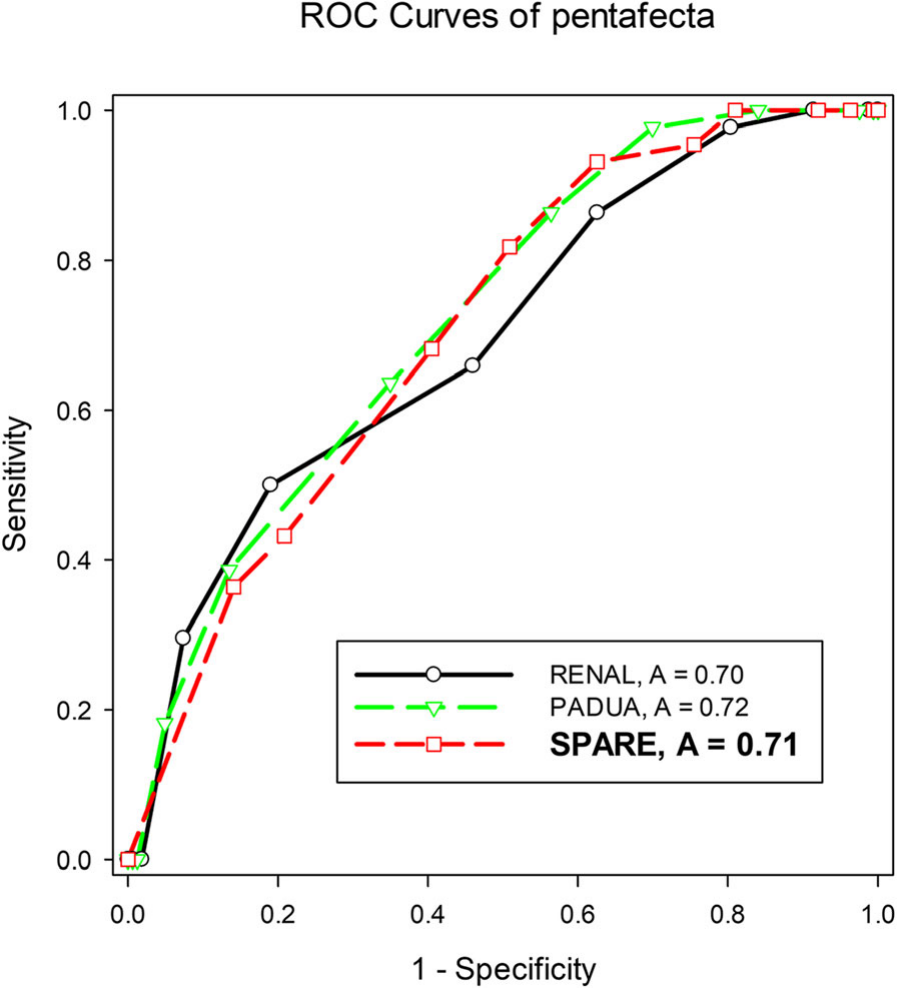
ROC curve analysis of pentafecta, the predictive ability of pentafecta was similar between nephrometries (SPARE vs. R.E.N.A.L., p = 0.78; SPARE vs. PADUA, p = 0.66)

Univariate analysis showed that sex (OR: 0.4, *p* = 0.006), BMI (OR: 0.92, p = 0.03) SPARE (OR: 0.66, *p* < 0.001), R.E.N.A.L. (OR: 0.65, *p* < 0.001), and PADUA (OR: 0.64, *p* < 0.001) significantly affected the achievement of pentafecta (Table 4). Multivariable models for the achievement of pentafecta using the variables with *p*-value below 0.25 from the univariable models are seen in Table 5. Regression analysis showed that all three nephrometries were independent predictive factors of pentafecta in each model (SPARE (OR: 0.67, *p* < 0.001), R.E.N.A.L. (OR: 0.66, *p* < 0.001), PADUA (OR:0.66, *p* < 0.001; Table 5). ROC analysis of pentafecta showed the fair predictive ability of the three nephrometries (SPARE (AUC: 0.71), R.E.N.A.L. (AUC: 0.7), PADUA (AUC: 0.72); Fig. 2). The predictive ability of pentafecta was similar between nephrometries (SPARE vs. R.E.N.A.L., *p* = 0.78; SPARE vs. PADUA, *p* = 0.66). The interobserver concordance between two urologists and one radiologist was almost perfect in total score (Kappa:0.89, *p* = 0.03), and in each component except for renal sinus involvement (Kappa: 0.69, *p* = 0.05) which was substantial (Table 6).

**Table 4:** Univariable model of pentafecta

	OR	95%CI	*P*-value
Age	1	(0.98, 1.03)	0.9
Sex	0.4	(0.2, 0.77)	0.006
ASA	0.84	(0.48, 1.47)	0.55
CCI	1.02	(0.88, 1.19)	0.81
BMI (kg/m^2^)	0.92	(0.84, 0.99)	0.03
Hypertension	1.71	(0.9, 3.9)	0.21
Diabetes	0.91	(0.44,1.6)	0.61
Pre-operative eGFR (ml/min/1.73m^2^)	1	(0.99, 1.01)	0.88
Surgical approach	1.48	(0.98, 2.25)	0.06
SPARE	0.66	(0.56, 0.79)	< 0.001
R.E.N.A.L.	0.65	(0.53, 0.81)	< 0.001
PADUA	0.64	(0.51, 0.79)	< 0.001

*ASA* American Society of Anesthesiologists score, *CCI* Charlson Comorbidity Index, *BMI* body mass index

**Table 5:** Multivariable model of pentafecta

	Model including SPARE			Model including RENAL			Model including PADUA		
	OR	95%CI	*P*-value	OR	95%CI	*P*-value	OR	95%CI	*P*-value
Sex	0.41	(0.2, 0.85)	0.02	0.39	(0.19, 0.8)	0.01	0.46	(0.23, 0.95)	0.04
BMI	0.9	(0.82, 0.98)	0.02	0.92	(0.85, 1)	0.07	0.91	(0.84, 1)	0.04
Surgical approach	1.46	(0.91, 2.36)	0.12	1.45	(0.92, 2.29)	0.1	1.47	(0.93, 2.32)	0.1
SPARE	0.67	(0.56, 0.8)	< 0.001						
R.E.N.A.L.				0.66	(0.53, 0.83)	< 0.001			
PADUA							0.66	(0.53, 0.82)	< 0.001

*ASA* American Society of Anesthesiologists score, *BMI* body mass index

**Table 6:** Interobserver concordance of the SPARE and PADUA system

	Kappa	P-value
SPARE score	0.89	0.03
Tumor size	0.93	< 0.001
Exophytic rate	0.87	0.02
Renal sinus involvement	0.69	0.05
Rim location	0.95	< 0.001
PADUA	0.71	0.04
Urinary collecting system	0.65	0.07
Longitudinal Polar location	0.68	0.2

## Discussion

Achieving trifecta and pentafecta is the major goal of PN regardless of the surgical approach. Therefore, an effective and validated tool to evaluate tumor complexity and surgical difficulty is essential. However, the R.E.N.A.L. and PADUA systems are not without limitations [10, 14]. The SPARE system, a refined version of PADUA, includes tumor size, exophytic rate, sinus involvement, and rim location (Fig. 1). Compared to R.E.N.A.L. and PADUA, the SPARE system had similar predictive ability in pentafecta achievement (Fig. 2). In other words, the fewer constituents of the SPARE system did not affect its efficacy while making it easier to calculate the score. Moreover, the interobserver concordance of the SPARE system was good in overall score and in most of the individual components (Table 6). As a result, the SPARE system appears to be a favorable choice when evaluating tumor complexity and predicting post-PN outcomes during clinical practice and patient counseling.

Most peri-operative outcomes in our study were similar to the RECORd1 project, a 4-year prospective observational multicenter study. The major complication rate was 3.5%, positive surgical margin rate was 5.5%, and median ischemia time was 16 min in the RECORd1 project [15, 16]. The longer median ischemia time (24 min) in our study may be due to larger tumor size and low volume center (less than 50 PN performed per year). Renal functional outcomes such as ACE at 3rd day and 30th day were similar between our study and the RECORd1 project.

Current study revealed that surgical approach were correlated to complication rates (ρ = − 0.23, *p* = 0.001), ischemia time (ρ = − 0.33, *p* < 0.001) but not with positive surgical margin (ρ = − 0.03, *p* = 0.76), PCE (ρ = − 0.06, *p* = 0.36) nor with achievement of pentafecta (ρ = 0.08, *p* = 0.23) (data not shown in tables). RECORd1 project mentioned that the open surgical approach was a significant predictive factor of complications. In contrast, Serni et al. showed that surgical approach was neither the predictor of trifecta outcome in patients with highly complex renal tumor underwent simple enucleation [17]. The effect of open surgical approach on trifecta/ pentafecta outcomes varied between studies may be caused by different surgical technique and different complexity of renal tumor. Further studies are required to confirm this hypothesis.

In the current study, SPARE nephrometry was correlated with peri-operative outcomes including ischemia time, operative time, and complication rate. RECORd1 project mentioned that modified PADUA is not an independent predictive factor of postoperative complications [15]. In contrast to the RECORd1 project, most patients in our cohort underwent standard PN by minimal invasive approach (76.8%). Since the utilization rate of open partial nephrectomy constantly decreased in last decades [16]. Therefore, SPARE would be a more suitable nephrometry in the era of minimally invasive surgery.

Although there was a trend toward greater functional loss in the higher risk group, Ficarra et al. found that the SPARE system was not associated with functional outcomes [13]. In contrast, the SPARE system was correlated with PCE and pentafecta in our study. This may be due to the different approaches of PN between the two studies. PN was conducted using standard resection methods in our institute, whereas 25% of the patients in their cohort underwent PN by enucleation [13]. Since resected renal volume plays an important role in functional loss [18], the predictive ability of the SPARE system in functional outcomes may be influenced by the volume of resected non-neoplastic renal parenchyma. In addition, tumor contact surface area has a greater ability to predict post-operative renal function than R.E.N.A.L. and PADUA [11, 19]. SPARE includes components such as radius (R) and exophytic rate (E), which is similar to tumor contact surface area [11]. The other two components of sinus involvement and rim location are related to the vascular territory of the kidneys which affect renal function deterioration [20]. As a result, the SPARE system may be correlated to functional outcomes to some extent. However, further well-designed studies are needed to confirm these hypotheses.

In our study, both R.E.N.A.L. and PADUA had a good predictive ability for pentafecta achievement. R.E.N.A.L. has been confirmed to be an independent predictive factor of pentafecta achievement with a negative association [21]. Serni et al. showed that PADUA score was significantly associated with the achievement of trifecta and with a negative margin, but not with warm ischemia time [17]. In contrast, Ubrig et al. and Harke et al. reported conflicting results about the predictive ability of PADUA for trifecta achievement [22, 23]. The difference regarding the predictive ability of PADUA in pentafecta achievement between studies may be explained by the following reasons. First, there were inconsistencies between studies in controlling for confounding factors such as comorbidities, and patient factors affect post-operative complication rates and functional change [6]. Differences in the methods of multivariate analysis between studies may have resulted in conflicting results. In our study, we included possible factors including age, Charlson Comorbidity Index, BMI, and pre-operative renal function in order to reduce selection bias. Second, unimportant and non-concordant factors in PADUA and a lack of central image review may have led to the difference in results between studies [24]. Third, different surgical approaches such as open/ laparoscopy/ robot or simple enucleation/ enucleoresection varied between studies may affect the pentafecta achievement.

Our study showed good interobserver concordance with the SPARE system, with a kappa value of 0.82. Hew et al. reported the limited reproducibility of the PADUA score [25], and they reported a Fleiss’ generalized kappa in their study cohort of 0.37 to 0.80 for the various components of the PADUA. Spaliviero et al. directly compared interobserver concordance among R.E.N.A.L., PADUA, and C-index, and found that agreement using the C-index method was higher than with PADUA or R.E.N.A.L. [24]. However, limitations existed when scoring the constituents including location and involvement of the collecting system [24]. Therefore, Ficarra et al. refined PADUA into the SPARE system which successfully improved interobserver agreement according to our results. In our cohort, the interobserver concordance of renal sinus involvement was lower and the exophytic rate was higher compared with previous studies. This may be because exophytic rate is a semi-quantitative parameter while renal sinus involvement is a qualitative parameter.

To the best of our knowledge, the current study is the first to externally validate the SPARE system. We further confirmed that SPARE is not only a predictive factor in overall complication rate, but also in pentafecta achievement. Besides complication rate, we also found similar predictive abilities of pentafecta achievement between the SPARE and R.E.N.A.L./PADUA systems in ROC analysis. Another strength of the current study is that we provided evidence of the reproducibility of the SPARE system between urologists and radiologist. This result suggests that the SPARE system can be applied across different specialties. However, there are also limitations to this study. First, this is a single center retrospective study design with various confounding factors. However, we tried our best to reduce selection bias by including possible confounding factors which have previously been reported. Second, we lacked unified imaging protocols for CT and MRI because we are a tertiary referral center. Most constituents of the SPARE system are quantitative or semi-quantitative, so there may not have been significant inconsistencies in the scoring. Third, only a small proportion of the patients (6.3%) were classified as being at high risk, which may have limited the findings. Fourth, the PN technique used in the current study was standard resection, so the applicability of SPARE for PN with enucleation is still unclear, and further studies are needed to confirm the efficacy of the SPARE system in high-risk renal tumors and PN with enucleation. Finally, we did not evaluate renal function using radio-isotope scans, which has been proven to be a more precise tool than serum Cre or eGFR [26], because the aim of this study was to assess pentafecta as defined by a change in renal function as assessed by eGFR [5]. This may not have limited the interpretation of the results.

## Conclusions

In conclusion, the results of this study showed that the SPARE system was a predictive factor of surgical outcomes after PN. This refined nephrometry had similar predictive abilities for pentafecta achievement compared with R.E.N.A.L. and PADUA. The reproducibility, efficacy, and ease of use mean that the SPARE system may replace R.E.N.A.L. and PADUA in clinical practice.

## Data Availability

The data supporting the conclusions are contained within the manuscript. The datasets used and analyzed during the current study are available from the corresponding author on reasonable request.

## Abbreviations

PN: Partial nephrectomy
eGFR: estimated glomerular filtration rates
SPARE: Simplified PADUA REnal nephrometry system
CT: Computed tomography
MRI: Magnetic resonance imaging
ACE: Absolute change of eGFR
PCE: Percent change of eGFR

## References

[1] Ljungberg B, Albiges L, Bensalah K, Bex A, Giles RH, Hora M, Kuczyk MA, Lam T, Marconi L, Merseburger AS (2018). EAU guidelines on renal cell carcinoma 2018. In: European Association of Urology guidelines 2018 edition. Volume presented at the EAU annual congress Copenhagen 2018, edn.

[2] Mir MC, Pavan N, Capitanio U, Antonelli A, Derweesh I, Rodriguez-Faba O, Linares E, Takagi T, Rha KH, Fiori C (2020). Partial versus radical nephrectomy in very elderly patients: a propensity score analysis of surgical, functional and oncologic outcomes (RESURGE project). World J Urol.

[3] Mir MC, Derweesh I, Porpiglia F, Zargar H, Mottrie A, Autorino R (2017). Partial nephrectomy versus radical nephrectomy for clinical T1b and T2 renal tumors: a systematic review and meta-analysis of comparative studies. Eur Urol.

[4] Hung AJ, Cai J, Simmons MN, Gill IS (2013). "trifecta" in partial nephrectomy. J Urol.

[5] Zargar H, Allaf ME, Bhayani S, Stifelman M, Rogers C, Ball MW, Larson J, Marshall S, Kumar R, Kaouk JH (2015). Trifecta and optimal perioperative outcomes of robotic and laparoscopic partial nephrectomy in surgical treatment of small renal masses: a multi-institutional study. BJU Int.

[6] Cacciamani GE, Gill T, Medina L, Ashrafi A, Winter M, Sotelo R, Artibani W, Gill IS (2018). Impact of host factors on robotic partial nephrectomy outcomes: comprehensive systematic review and meta-analysis. J Urol.

[7] Kutikov A, Uzzo RG (2009). The R.E.N.a.L. nephrometry score: a comprehensive standardized system for quantitating renal tumor size, location and depth. J Urol.

[8] Ficarra V, Novara G, Secco S, Macchi V, Porzionato A, De Caro R, Artibani W (2009). Preoperative aspects and dimensions used for an anatomical (PADUA) classification of renal tumours in patients who are candidates for nephron-sparing surgery. Eur Urol.

[9] Schiavina R, Novara G, Borghesi M, Ficarra V, Ahlawat R, Moon DA, Porpiglia F, Challacombe BJ, Dasgupta P, Brunocilla E (2017). PADUA and R.E.N.a.L. nephrometry scores correlate with perioperative outcomes of robot-assisted partial nephrectomy: analysis of the Vattikuti global quality initiative in robotic urologic surgery (GQI-RUS) database. BJU Int.

[10] Ficarra V, Crestani A, Bertolo R, Antonelli A, Longo N, Minervini A, Novara G, Simeone C, Carini M, Mirone V (2019). Tumour contact surface area as a predictor of postoperative complications and renal function in patients undergoing partial nephrectomy for renal tumours. BJU Int.

[11] Hsieh PF, Wang YD, Huang CP, Wu HC, Yang CR, Chen GH, Chang CH (2016). A mathematical method to calculate tumor contact surface area: an effective parameter to predict renal function after partial nephrectomy. J Urol.

[12] Watts KL, Ghosh P, Stein S, Ghavamian R (2017). Value of Nephrometry score constituents on perioperative outcomes and Split renal function in patients undergoing minimally invasive partial nephrectomy. Urology..

[13] Ficarra V, Porpiglia F, Crestani A, Minervini A, Antonelli A, Longo N, Novara G, Giannarini G, Fiori C, Simeone C, et al. The simplified PADUA REnal (SPARE) nephrometry system: a novel classification of parenchymal renal tumours suitable for partial nephrectomy. BJU Int. 2019;124:621–28. 10.1111/bju.14772 30963680

[14] Khene ZE, Peyronnet B, Kocher NJ, Robyak H, Robert C, Pradere B, Oger E, Kammerer-Jacquet SF, Verhoest G, Rioux-Leclercq N (2018). Predicting morbidity after robotic partial nephrectomy: The effect of tumor, environment, and patient-related factors. Urol Oncol.

[15] Mari A, Antonelli A, Bertolo R, Bianchi G, Borghesi M, Ficarra V, Fiori C, Furlan M, Giancane S, Longo N (2017). Predictive factors of overall and major postoperative complications after partial nephrectomy: results from a multicenter prospective study (the RECORd 1 project). Eur J Surg Oncol.

[16] Schiavina R, Mari A, Antonelli A, Bertolo R, Bianchi G, Borghesi M, Brunocilla E, Fiori C, Longo N, Martorana G (2015). A snapshot of nephron-sparing surgery in Italy: a prospective, multicenter report on clinical and perioperative outcomes (the RECORd 1 project). Eur J Surg Oncol.

[17] Serni S, Vittori G, Frizzi J, Mari A, Siena G, Lapini A, Carini M, Minervini A (2015). Simple enucleation for the treatment of highly complex renal tumors: perioperative, functional and oncological results. Eur J Surg Oncol.

[18] Klingler MJ, Babitz SK, Kutikov A, Campi R, Hatzichristodoulou G, Sanguedolce F, Brookman-May S, Akdogan B, Capitanio U, Roscigno M (2019). Assessment of volume preservation performed before or after partial nephrectomy accurately predicts postoperative renal function: results from a prospective multicenter study. Urol Oncol.

[19] Wang YD, Huang CP, Chang CH, Wu HC, Yang CR, Wang YP, Hsieh PF (2019). The role of RENAL, PADUA, C-index, CSA nephrometry systems in predicting ipsilateral renal function after partial nephrectomy. BMC Urol.

[20] Masago T, Yamaguchi N, Iwamoto H, Morizane S, Hikita K, Honda M, Sejima T, Takenaka A (2018). The significance of predictable traumatic area by renorrhaphy in the prediction of postoperative ipsilateral renal function. Cent European J Urol.

[21] Kahn AE, Shumate AM, Ball CT, Thiel DD (2020). Pre-operative factors that predict trifecta and pentafecta in robotic assisted partial nephrectomy. J Robot Surg.

[22] Ubrig B, Roosen A, Wagner C, Trabs G, Schiefelbein F, Witt JH, Schoen G, Harke NN (2018). Tumor complexity and the impact on MIC and trifecta in robot-assisted partial nephrectomy: a multi-center study of over 500 cases. World J Urol.

[23] Harke NN, Mandel P, Witt JH, Wagner C, Panic A, Boy A, Roosen A, Ubrig B, Schneller A, Schiefelbein F (2018). Are there limits of robotic partial nephrectomy? TRIFECTA outcomes of open and robotic partial nephrectomy for completely endophytic renal tumors. J Surg Oncol.

[24] Spaliviero M, Poon BY, Aras O, Di Paolo PL, Guglielmetti GB, Coleman CZ, Karlo CA, Bernstein ML, Sjoberg DD, Russo P (2015). Interobserver variability of R.E.N.a.L., PADUA, and centrality index nephrometry score systems. World J Urol.

[25] Hew MN, Baseskioglu B, Barwari K, Axwijk PH, Can C, Horenblas S, Bex A, Rosette JJ, Pes MP (2011). Critical appraisal of the PADUA classification and assessment of the R.E.N.a.L. nephrometry score in patients undergoing partial nephrectomy. J Urol.

[26] Zargar H, Akca O, Autorino R, Brandao LF, Laydner H, Krishnan J, Samarasekera D, Stein RJ, Kaouk JH (2015). Ipsilateral renal function preservation after robot-assisted partial nephrectomy (RAPN): an objective analysis using mercapto-acetyltriglycine (MAG3) renal scan data and volumetric assessment. BJU Int.

